# Squamous cell carcinoma arising from an unknown primary site metastasizing to the stomach: A case report

**DOI:** 10.3892/ol.2014.1836

**Published:** 2014-01-28

**Authors:** GUANGYAO WANG, PING CHEN, LEI SHI, WEI ZHAO

**Affiliations:** Department of Gastrointestinal Surgery, Subei People’s Hospital of Jiangsu Province, Yangzhou University, Yangzhou, Jiangsu 225001, P.R. China

**Keywords:** squamous cell carcinoma, metastasis, gastric carcinoma

## Abstract

Primary squamous cell carcinoma (SCC) may originate elsewhere in the body, including the head, neck, lung, bronchus, cervix uteri, esophagus and cardia, and metastasize to the stomach. In the present report, a case is presented of an SCC, which arose from an unknown primary site and metastasized to the stomach of a 59-year-old male. The tumor was located in the interspace between the liver and the stomach. It involved the placenta percreta, lamina muscularis and submucosa, however, had already metastasized to a regional lymph node at the time of surgery. No SCC was observed in other organs on physical examination, which included positron emission tomography-computed tomography. In the follow-up period, there was no evidence of additional malignant tumors in the patient; therefore, the origin of the tumor was speculative. To the best of our knowledge, this is the first case report regarding a tumor of this type.

## Introduction

Primary squamous cell carcinoma (SCC) arising from an unknown primary site and metastasizing to the stomach is extremely rare (1–9). Due to the poor prognosis associated with primary SCC and the requirement for an appropriate diagnosis, special attention is required that may lead to an improved therapeutic strategy. In the present study, a unique case of an SCC in the interspace between the liver and stomach is reported. Furthermore, the present report identifies the possible pathogenesis, diagnosis and treatment of this type of tumor. The study was approved by the ethics committee of Subei People’s Hospital of Jiangsu Province (Yangzhou, China) and the patient provided consent for publication.

## Case report

A 59-year-old male was admitted to Subei People’s Hospital of Jiangsu Province on June 21, 2012, complaining of upper abdominal pain with no evident inducible factor, which had persisted for one month; the pain was discontinuous and dull. The patient’s past medical history was unremarkable, with the exception of mild weight loss. The patient had smoked one pack of cigarettes per day for 25 years. A physical examination revealed no abdominal mass; however, there was tenderness in the right upper quadrant of the abdomen.

**Figure 1:**
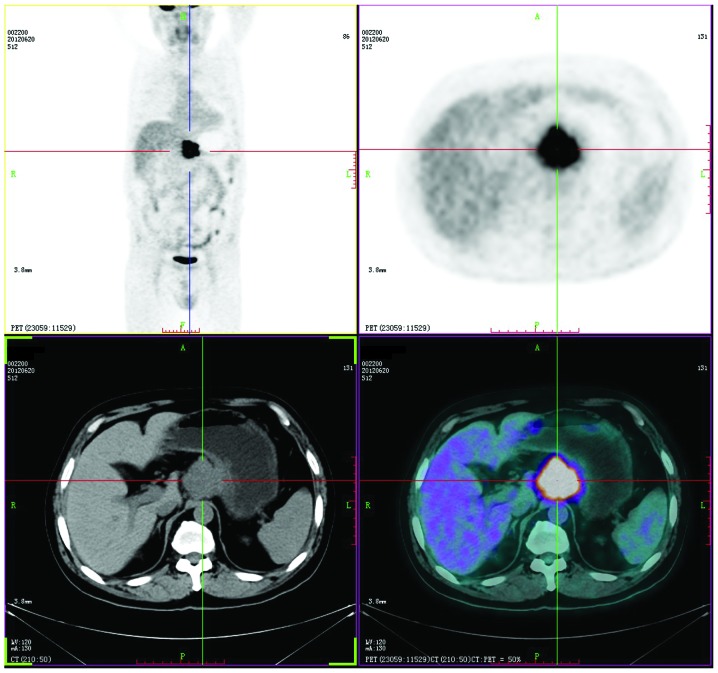
Positron emission tomography-computed tomography demonstrated a hypermetabolic lesion (standardized uptake value, 12.8) in the interspace between the liver and stomach. There was no additional fludeoxyglucose (18F) uptake, which indicated a primary site in other organs.

**Figure 2:**
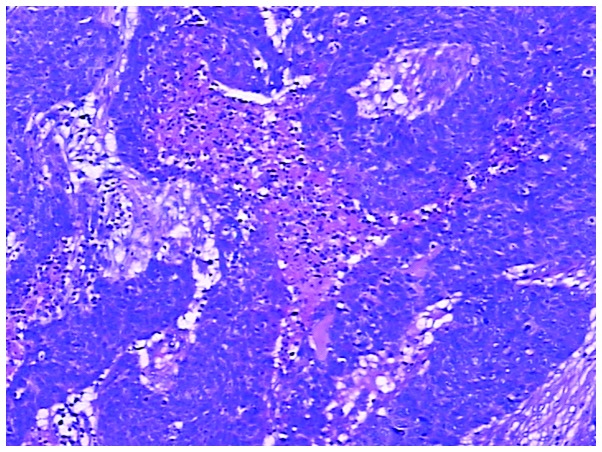
Intraoperative fast pathological sections revealed that the tumor tissues were composed of nidulant, multi-mitotic cells and necrosis, which was reported as a squamous cell carcinoma. (Hematoxylin and eosin staining; magnification, ×100).

Laboratory studies revealed that the complete blood count and blood chemistry were within the normal range. No abnormal tumor markers were detected: Cancer antigen (CA)125, 3.15 KU/l (<35.00 KU/l); CA15-3, 2.16 KU/l (<35.00 KU/l); CA19-9, 3.57 KU/l (<35.00 KU/l); CA242, 2.58 KU/l (<20.00 KU/l); α-fetoprotein, 6.57 ng/ml (<20.00 ng/ml); carcinoembryonic antigen, 2.25 ng/ml (<5.00 ng/ml); neuron-specific enolase, <1.0 ng/ml (<13.00 ng/ml); ferritin, 19.70 ng/ml (male, <322.00 ng/ml; female, <219.00 ng/ml); human growth hormone, 2.20 ng/ml (<7.50 ng/ml); or β-human chorionic gonadotropin, <0.03 mIU/ml (<3.00 mIU/ml). Axial positron emission tomography (PET), computed tomography (CT), PET/CT and maximum intensity projection images are shown in Fig. 1. PET/CT identified a hypermetabolic lesion in the interspace between the liver and stomach. There was no additional fludeoxyglucose (18F) uptake, which indicated a primary site on the transaxial PET/CT scans of the head, neck, chest, pelvis, extremities or other abdominal organs (including the spleen, pancreas, gallbladder, kidney, large and small intestines). The patient subsequently underwent surgical resection. The tumor was located in the interspace between the liver and stomach, it measured 6×5 cm, was close to the left gastric artery, and invaded the gastric wall and the pancreas. Intraoperative fast pathological sections revealed that the tumor tissues were composed of nidulant, multi-mitotic cells and necrosis, which were identified as SCC (Fig. 2); therefore, the tumor was resected and a proximal gastrectomy was performed.

**Figure 3:**
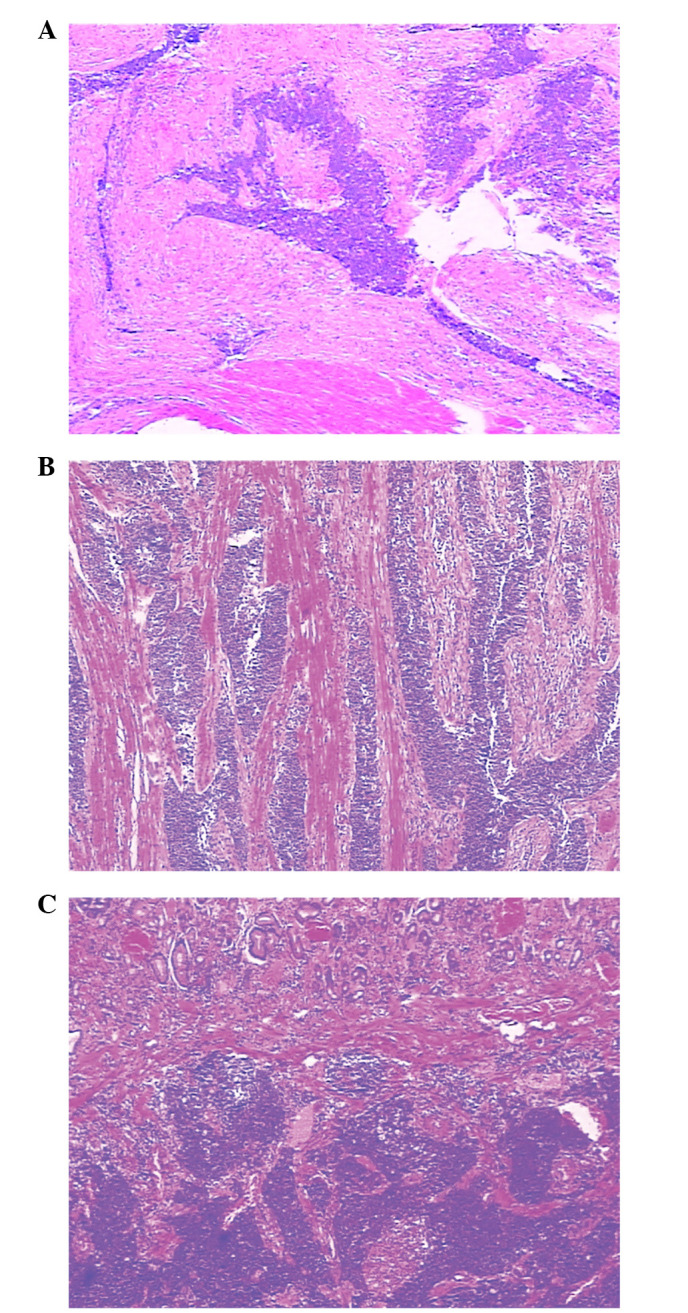
Tumor cells were observed in the (A) gastric serosa fibrous tissue, (B) lamina muscularis and (C) submucosa. (Hematoxylin and eosin staining; magnification, ×100).

**Figure 4:**
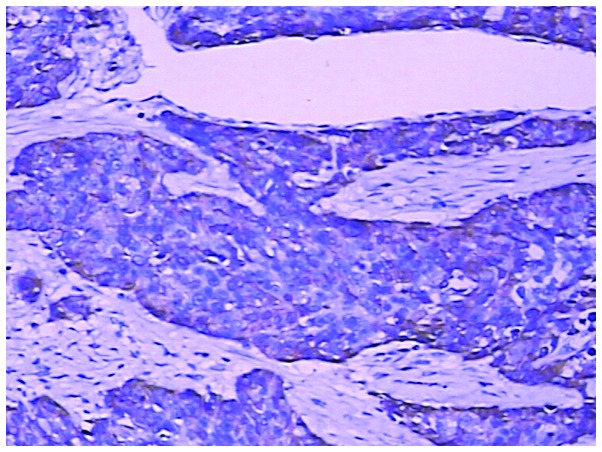
Strong expression of cytokeratin 5/6 observed via immunohistochemical staining. (Hematoxylin and eosin staining; magnification, ×200).

**Figure 5:**
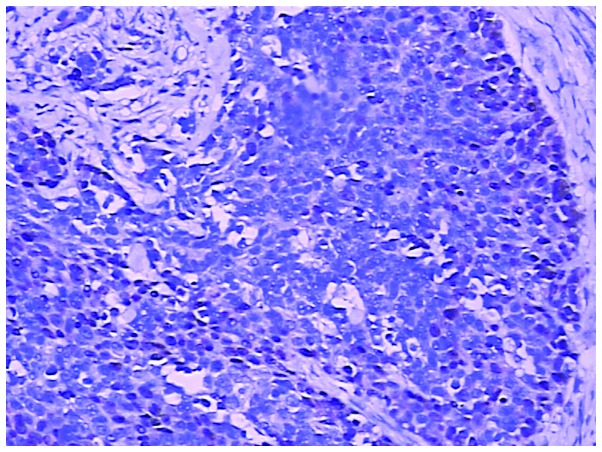
Immunohistochemical staining identified p63 positivity in the tumor cells. (Hematoxylin and eosin staining; magnification, ×200).

**Figure 6:**
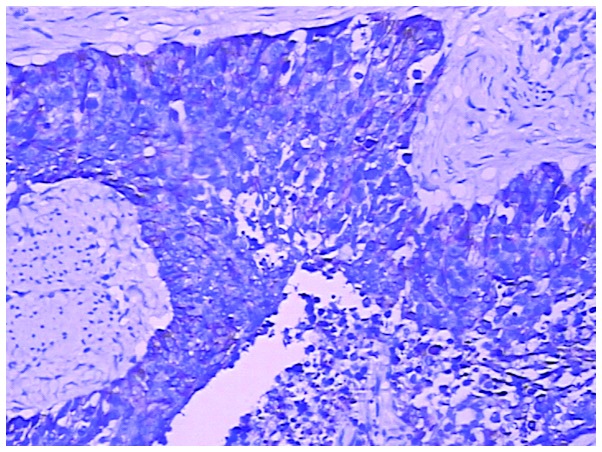
Immunohistochemical staining identified cytokeratin pan positivity in the tumor cells. (Hematoxylin and eosin staining; magnification, ×200).

**Figure 7:**
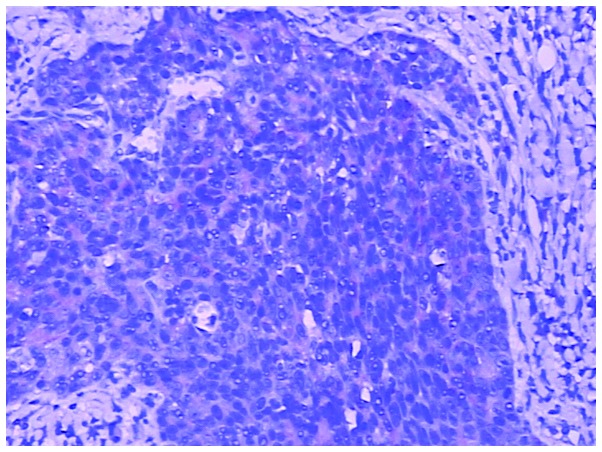
Positive glutathione S-transferase π immunostaining was observed in the neoplastic cells. (Hematoxylin and eosin staining; magnification, ×200).

**Figure 8:**
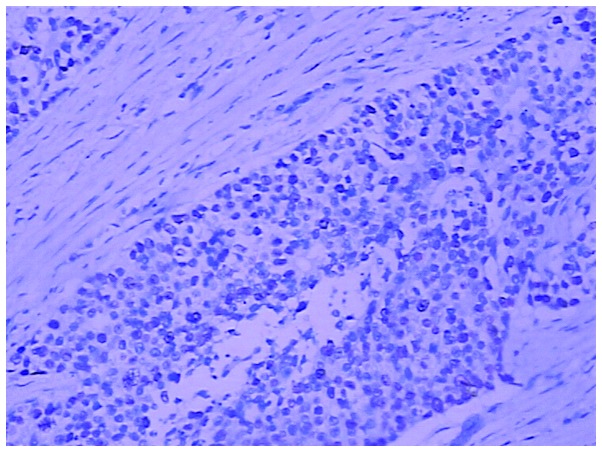
Ki-67 proliferation index, ~50%. (Hematoxylin and eosin staining; magnification, ×200).

Histopathology revealed that the SCC infiltrated into the serosal fibrous tissue, lamina muscularis and submucosa of the gastric wall (Fig. 3A–C); however, there was no cancer cell infiltration into the mucosa. The surgical margins were negative, however, metastasis to one lymph node in the lesser curvature of the stomach was observed. Furthermore, the esophagogastric junction was negative for the tumor. The immunohistochemical staining was positive for cytokeratin (CK)5/6, p63, CKpan and glutathione S-transferase π (Figs. 4–7), and negative for cluster of differentiation (CD)56, CDX-2, chromogranin A, CK20, CK7, S100, Syn, Villin, P-glycoprotein, epidermal growth factor receptor, topoisomerase II and p53. The Ki-67 proliferation index was ~50% (Fig. 8). Due to the progression of the disease, postoperative chemotherapy was recommended, and the patient and the patient’s family consented to four sessions of chemotherapy. At the end of the 12-month follow-up, which was conducted using ultrasonography and CT, the patient had survived and there was no evidence of recurrence and metastasis.

## Discussion

Primary SCC of the stomach was first described in 1895 (10) and remains a rare entity. SCC occurs mostly in males (male to female ratio, 5:1) (11). The diagnostic criteria for primary SCC of the stomach are as follows: The tumor must not be located in the cardia; the tumor must not extend into the esophagus; and there must be no evidence of SCC in any other organs. In the present case, the cardia was intact and the 5-cm segment between the esophagus and the proximal portion of the tumor was normal. No SCC findings were determined in other organs during the physical examination. Throughout the follow-up period, there was no evidence of other malignant tumors in the patient. Boswell and Helwig (12) defined four histopathological criteria that are required for the diagnosis of SCC of the stomach: Presence of keratinizing cell masses with pearl formation, a mosaic cell arrangement, intracellular bridges and high concentrations of sulfhydryl or disulfide bonds, which indicate keratin production.

To reach a diagnosis of primary SCC of the stomach, alternative sources of malignant squamous cells must be excluded. For example, islands of squamous cells originating from the esophagus may exist in the cardia and may be a potential source for the development of SCC. SCCs that originate in the esophagus itself may extend into the stomach by direct invasion. Primary SCC may originate elsewhere in the body (for example, the head, neck, lung, bronchus and cervix uteri) and metastasize to the stomach. Metastatic disease from such sites may be ruled out by clinical examination, particularly PET/CT.

Usually, primary SCC of the stomach initially invade the mucosa; those limited to outside the area of the mucous membrane are extremely rare. Histologically, the tumor tissues were located outside the area of the mucous membrane without adenoid tumor tissue structures or tumor mucosal lesions; therefore, it may be considered that the tumor tissues were not associated with the mucosal epithelium and glands. Therefore, the present case is neither primary SCC nor adenoacanthoma of the stomach. Furthermore, gross and pathological examinations eliminated direct invasion of the esophageal SCC into the stomach. No tumors were determined in other organs during the physical examination that included PET/CT. Therefore, the tumor was identified as a metastatic gastric tumor or an SCC arising from an unknown primary site and metastasizing to the stomach.

In a previous study, immunohistochemical staining showed strong staining for high molecular weight CK5/6 and p63 in the SCC (1). The immunohistochemical findings of the case in the present study were consistent with these. Therefore, immunohistochemical examination should be performed and immunomarkers, including CK5/6 and p63, should be included in the immunohistochemistry examination.

Since the tumor is extremely uncommon, the mechanism has not been well elucidated. Numerous pathological processes have been proposed, including heterotopic squamous epithelium, squamous metaplasia, multipotent stem cells capable of differentiating into cells of any type, overgrowth of the squamous component in primary adenocarcinoma and local extension or metastasis of esophageal SCC (13). The favored suggestion is the malignant transformation in an area of squamous metaplasia. The development of SCC in the absence of metaplasia, but with chronic inflammation, has previously been reported (1). Straus *et al* (14) performed detailed histological examinations of patients that were considered to have pure SCC and demonstrated the presence of glandular components. Mori *et al* (13) proposed the following hypothesis based on these findings: Neoplastic multipotent cells initially become adenocarcinomas, which is followed by the occurrence of squamous metaplasia and subsequent conversion into SCC.

Owing to the rarity and advanced presentation of SCC, there is a lack of evidence to support a particular management strategy and prognosis is difficult to predict. Surgery to achieve R0 (no residual tumor) resection remains the mainstay of the treatment and is well supported by adjuvant therapies as reported in the present study. Regardless of undergoing only four chemotherapy sessions, the patient in the present study did not exhibit recurrence or metastasis; therefore, paclitaxel and platinum-based agents are recommended. A good response to chemoradiotherapy, with respect to recurrence, following surgical resection was reported by Michalet *et al* (15). Adjuvant radiotherapy following surgical resection and subsequent chemotherapy have resulted in survival periods of >3 years, in a case of advanced stage SCC the patient was free of recurrence with a good quality of life for five years (2). Good long-term survival in gastric SCC has been reported with chemotherapy alone despite locally invasive tumors (3). However, insufficient information is available on the adjuvant role of chemoradiotherapy in SCC arising from an unknown primary site metastasizing to the stomach compared with other digestive tumors, such as esophageal SCC, where it has been shown to have a definite role. Its pathogenesis, diagnosis and treatment remain a topic of debate and further studies would benefit the patients that are affected by this rare disease.

In conclusion, a rare case of SCC from an unknown primary site metastasizing to the stomach is presented. To the best of our knowledge, this is the first case report to have investigated this tumor type. The pathogenesis, diagnosis and treatment of an SCC of this type has generally been considered poor, thus, additional cases require investigation to confirm this.
